# The female epilepsy protein PCDH19 is a new GABA_A_R-binding partner that regulates GABAergic transmission as well as migration and morphological maturation of hippocampal neurons

**DOI:** 10.1093/hmg/ddy019

**Published:** 2018-01-17

**Authors:** Silvia Bassani, Andrzej W Cwetsch, Laura Gerosa, Giulia M Serratto, Alessandra Folci, Ignacio F Hall, Michele Mazzanti, Laura Cancedda, Maria Passafaro

**Affiliations:** 1 CNR Institute of Neuroscience, Milan 20129, Italy; 2 Department of Neuroscience and Brain Technologies, Istituto Italiano di Tecnologia, Genoa 16163, Italy; 3 Department of Bioscience, University of Milan, Milan 20133, Italy; 4 Telethon Dulbecco Institute, Milan, Italy

## Abstract

The *PCDH19* gene (Xp22.1) encodes the cell-adhesion protein protocadherin-19 (PCDH19) and is responsible for a neurodevelopmental pathology characterized by female-limited epilepsy, cognitive impairment and autistic features, the pathogenic mechanisms of which remain to be elucidated. Here, we identified a new interaction between PCDH19 and GABA_A_ receptor (GABA_A_R) alpha subunits in the rat brain. PCDH19 shRNA-mediated downregulation reduces GABA_A_R surface expression and affects the frequency and kinetics of miniature inhibitory postsynaptic currents (mIPSCs) in cultured hippocampal neurons. *In vivo*, PCDH19 downregulation impairs migration, orientation and dendritic arborization of CA1 hippocampal neurons and increases rat seizure susceptibility. In sum, these data indicate a role for PCDH19 in GABAergic transmission as well as migration and morphological maturation of neurons.

## Introduction

Mutations in the *PCDH19* gene on chromosome X (Xp22.1) cause a female-limited epilepsy (PCDH19 Female Epilepsy, PCDH19-FE; OMIM # 300088) that is frequently associated with intellectual disability and autistic features (1,2). Since the discovery of its involvement in PCDH19-FE in 2008, *PCDH19* has rapidly become the second most clinically relevant gene in epilepsy after the Dravet syndrome causative gene SCN1A (3).

*PCDH19* encodes protocadherin-19 (PCDH19), a calcium-dependent cell-adhesion molecule belonging to the non-clustered delta2-protocadherin subclass of the cadherin superfamily (4,5). PCDH19 is composed of six conserved extracellular cadherin (EC) repeats, a transmembrane region and an intracellular C-terminal tail with two conserved motifs, (CM)1 and CM2, specific for delta protocadherins (6). To date, more than 100 inherited or *de novo* mutations have been reported, including point mutations and partial or whole-gene deletions (5,7).

PCDH19 has been shown to have a role in migration and neural circuit formation in zebrafish (8,9) and to control the proliferation of neuronal progenitors in mouse cortex (10). Despite these findings, a comprehensive understanding of the biological function of *PCDH19* as well as its role in PCDH19-FE pathogenesis is lagging behind. Here, we report a new interaction between PCDH19 and GABA_A_ receptors (GABA_A_Rs) in neurons, suggesting the involvement of PCDH19 in regulating GABAergic transmission.

Malfunctioning of the GABAergic system, and in particular in GABA_A_ receptor (GABA_A_R)-mediated transmission, is a common hallmark of neurodevelopmental disorders associated with intellectual disability, epilepsy and autism (11,12). Indeed, defects in GABA_A_R currents predispose the brain to seizures and might leave a permanent mark that will result in cognitive impairment and behavioral problems (13). GABA_A_Rs are heteropentameric channels permeated by chloride and bicarbonate ions, composed of a combination of 19 subunits with different kinetics and pharmacology (14). Typically, GABA_A_Rs are composed of 2 alpha, 2 beta and 1 gamma or delta subunits. Receptors composed of alpha 1–3 or alpha 5 together with beta and gamma subunits are mainly synaptic and mediate phasic inhibition, with the exception of extrasynaptic alpha 5-containing receptors. Conversely, receptors composed of alpha 4 or 6 subunits together with beta and delta subunits are extrasynaptic and mediate tonic inhibition (15).

While in the adult brain GABA_A_Rs mediate fast inhibitory transmission, in the immature brain GABA is depolarizing and mostly excitatory due to the high intracellular chloride concentration, and regulates neuronal proliferation, migration and differentiation (12).

Here, we found that PCDH19 binds the alpha subunits of GABA_A_R and regulates its surface availability and currents in cultured hippocampal neurons. *In vivo*, PCDH19 downregulation throughout embryonic development and the first postnatal week affects neuronal migration and morphological maturation and increases seizure susceptibility. Altogether, our findings highlight PCDH19 as a key modulator of GABAergic transmission and suggest new pathogenic mechanisms for PCDH19-FE.

## Results

### PCDH19 interacts with GABA_A_R in neurons

Our study stems from the finding that PCDH19 is able to bind GABA_A_R both in heterologous cells and in rat neurons. Given the central role of GABA_A_R in epilepsy and comorbidities of neurodevelopmental disorders, we decided to investigate the PCDH19-GABA_A_R association in more detail.

**Figure 1.**
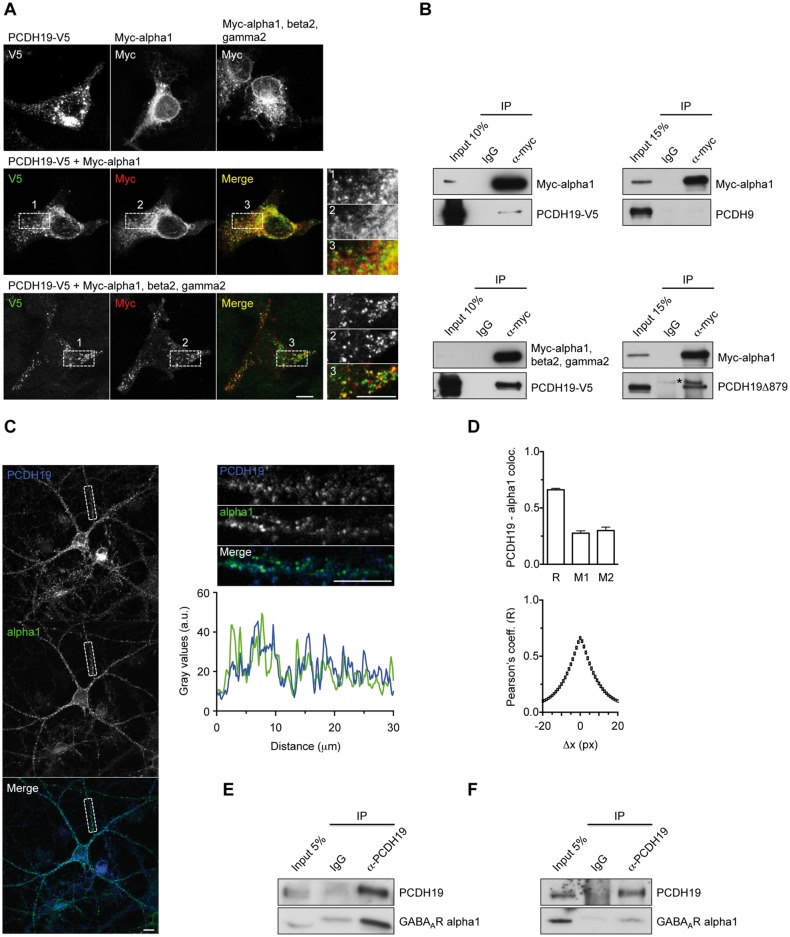
PCDH19 interacts with GABA_A_R alpha 1 subunit. (**A**) Confocal images of HEK293T cells transfected with PCDH19-V5, GABA_A_R myc-alpha 1 subunit, myc-alpha 1 plus beta 2 and gamma 2 subunits either alone (top panel) or in combination (middle and lower panel) and stained with anti-V5 and anti-myc antibody. PCDH19-V5 colocalizes with alpha 1 subunit, especially in the perinuclear region when alpha 1 is expressed alone (middle panel and relative inset magnifications) and mostly in vesicle-like clusters when alpha 1 is expressed together with beta 2 and gamma 2 subunits (lower panel and magnifications). The scale bar represents 10 μm. (**B**) CoIP in HEK293T cells expressing the GABA_A_R subunit myc-alpha 1 (alone or in combination with beta 2 and gamma 2) and either full-length PCDH19 (PCDH19-V5), truncated PCDH19 (PCDH19Δ879) or PCDH9. Both full-length and truncated PCDH19, but not PCDH9, were immunoprecipitated by anti-myc. The asterisk indicates a non-specific signal in both the IgG and anti-myc lanes. (**C**) Representative images of cultured rat hippocampal neurons double-stained for PCDH19 and alpha 1 at DIV18. The fluorescence intensity profiles plot refers to the dendrite in the magnification insets. The images represent single Z-sections. The scale bar represents 10 μm. (**D**) Summary of colocalization experiments. Pearson’s (R, 0.662 ± 0.013) and Manders’ (M1 = fraction of PCDH19 overlapping alpha 1, 0.276 ± 0.020 and M2 = fraction of alpha 1 overlapping PCDH19, 0.299 ± 0.031) coefficients (top) and Van Steensel's CCF (bottom), obtained by shifting the alpha 1 channel in the X-direction relative to the PCDH19 channel and plotting R as a function of Δx (pixel shift) (see Supplementary Material, Table S1). The sharp correlation decrease excludes random colocalization (bottom). (**E, F**) CoIP of endogenous PCDH19 and alpha 1 in cultured neurons (E) and brain homogenate (cortex plus hippocampus) (F).

We first observed the interaction in HEK293T cells expressing PCDH19 together with GABA_A_R alpha 1 subunit alone or together with beta 2 and gamma 2 subunits to allow the assembly of a pentameric receptor able to exit the endoplasmic reticulum (ER) and reach the cell surface (14). Immunocytochemistry (ICC) experiments revealed PCDH19-alpha 1 colocalization, which was most evident in the perinuclear region, likely the ER, when alpha was the only overexpressed GABA_A_R subunit, and in vesicle-like structures when alpha 1 was coexpressed with the other receptor subunits (Fig. 1A).

Moreover, alpha 1 expressed alone or with beta 2 and gamma 2 subunits in HEK293T cells, coimmunoprecipitated with PCDH19 but not with delta protocadherin PCDH9, which shares CM1 and CM2 regions with PCDH19, ruling out the contribution of these conserved regions in mediating the GABA_A_R interaction. Consistently, a PCDH19 mutant without the distal portion of the intracellular tail containing CM1 and CM2 (PCDH19Δ879) still coimmunoprecipitated with alpha 1 (Fig. 1B).

Next, we measured the extent of colocalization of the endogenous proteins in cultured hippocampal neurons (Fig. 1C and D; Supplementary Material, Table S1). Based on the analysis of ICC images, we estimated that approximately 30% of PCDH19 colocalized with alpha 1 (Fig. 1D; Supplementary Material, Table S1). Finally, an anti-PCDH19 antibody coimmunoprecipitated alpha 1 both in hippocampal neurons (Fig. 1E) and in brain homogenate (Fig. 1F). Therefore, we concluded that PCDH19 and the GABA_A_R subunit alpha 1 were associated in neurons.

### The PCDH19 C-terminus binds to a conserved region in the TM3–4 loop of GABA_A_R alpha subunits

To define the minimal region of interaction between PCDH19 and alpha 1, we performed a GST pull-down assay using selected protein domains fused to GST.

**Figure 2.**
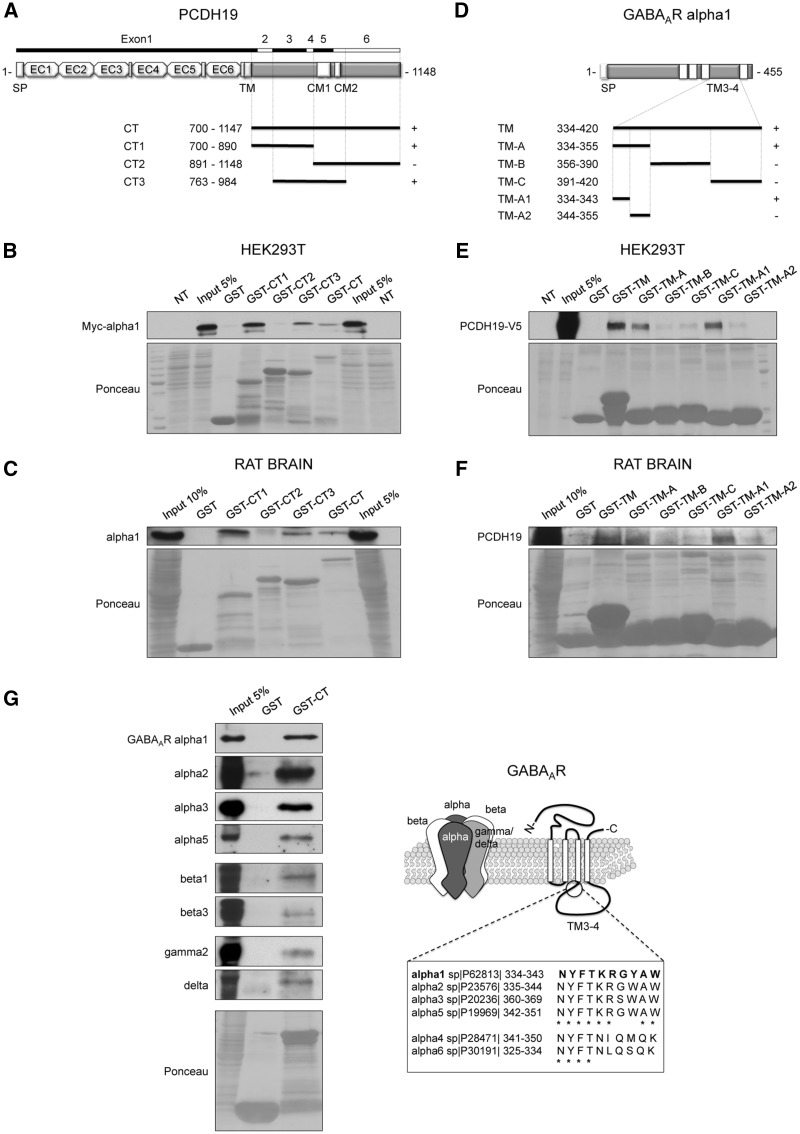
Mapping of the PCDH19-GABA_A_R-interacting region. (**A**) Schematic of the PCDH19 structure and C-terminus fragments that were fused to GST (CT, CT1, CT2, CT3) with relative amino acid extension. SP, signal peptide; TM, transmembrane domain; CM1 and CM2, conserved motifs 1 and 2. (**B**, **C**) GST pull-down assay showing that the PCDH19 constructs GST-CT, GST-CT1 and GST-CT3, but not GST-CT2, pulled-down myc-alpha 1 overexpressed in HEK293T cells (B) and endogenous alpha 1 in rat brain (C). (**D**) Schematic of the GABA_A_R subunit alpha 1 and intracellular loop TM3-4 fragments that were fused to GST with relative amino acid extension. (**E**, **F**) GST pull-down assay showing that the alpha 1 constructs GST-TM, GST-TM-A and GST-TM-A1, but not GST-TM-B, GST-TM-C and GST-TM-A2, pulled-down PCDH19-V5 overexpressed in HEK293T cells (E) and PCDH19 endogenously expressed in rat brain (F). (**G**) GST pull-down in rat brain (cortex plus hippocampus) using PCDH19 GST-CT (left) and cartoon showing the GABA_A_R subunit composition and structure and alignment of alpha 1 amino acids 334-343 with the corresponding amino acids in the other alpha subunits (right).

In particular, we tested the entire PCDH19 intracellular C-terminal tail (CT) and its proximal (CT1), distal (CT2) and central (CT3) region separately (Fig. 2A). We found that CT1 and CT3 in addition to CT, but not CT2, were able to pull-down alpha 1 either overexpressed in HEK293T cells (Fig. 2B) or endogenously expressed in rat brain (Fig. 2C). These data further ruled out the involvement of the protocadherin conserved motifs CM1 and CM2 in mediating the GABA_A_R interaction and suggested the 128 amino acids shared by CT1 and CT3 (amino acids 763–890) as those sufficient to bind alpha 1. Taking into account the coimmunoprecipitation data (Fig. 1B), the interacting region might be further restricted to amino acids 763–879.

Next, we repeated analogous experiments by focusing on the alpha 1 TM3–4 intracellular loop since this region mediates GABA_A_R intracellular interactions (14). We fused GST to the entire TM3–4 loop (TM) and to its three different fragments (TM-A, TM-B, TM-C) (Fig. 2D). GST-TM-A was sufficient to pull-down PCDH19-V5 overexpressed in HEK293T cells, so we further divided it in two (TM-A1 and TM-A2, Fig. 2D) and identified TM-A1 (amino acids 334–343) as the minimal region mediating the PCDH19 interaction (Fig. 2E). The GST pull-down assay using rat brain confirmed these results (Fig. 2F).

The TM-A1 sequence, corresponding to the first ten amino acids of the TM3–4 loop, is highly conserved in alpha subunits, especially in alpha 1–3 and 5 (Fig. 2G, right panel). Therefore, we verified whether PCDH19 could also associate with these alpha subunits in rat brain homogenate from cortex and hippocampus. As expected, PCDH19 GST-CT pulled down alpha 1–3 and 5 and their associated beta (beta 1 and 3) and gamma (gamma 2) subunits. In addition to alpha 5, the delta subunit was also pulled down, suggesting that the PCDH19 association with GABA_A_R might involve both synaptic and extrasynaptic receptors (Fig. 2G, left panel).

In conclusion, we found that the PCDH19 CT domain upstream of CM1 was able to bind the first 10 conserved amino acids of alpha subunits TM3–4.

### The PCDH19 level affects GABA_A_R surface expression and currents

The interaction between PCDH19 and GABA_A_R prompted us to investigate the consequences of altered PCDH19 expression on the composition and functioning of GABAergic synapses.

**Figure 3.**
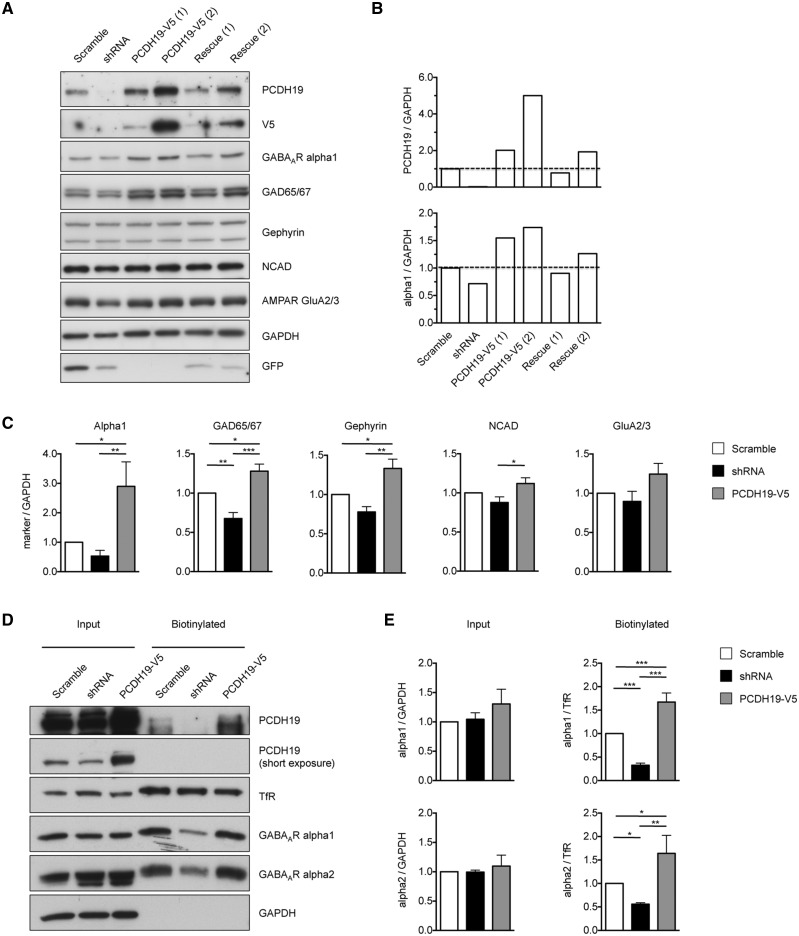
The PCDH19 expression level affects inhibitory markers and surface GABA_A_R expression. (**A**) Western blot of cultured neurons infected at DIV1 with lentiviruses expressing PCDH19 control shRNA (Scramble), shRNA, PCDH19-V5 [at two different concentrations: 1X (1) and 2X (2), where the 1X concentration is sufficient to infect approximately 80-100% of cells], or shRNA plus PCDH19-V5 (Rescue1 and 2, according to the PCDH19-V5 virus concentration used, either 1X or 2X) and lysed at DIV8. Anti-PCDH19 detects both endogenous and lentivirally expressed protein, while anti-V5 detects exclusively the lentivirally expressed protein. Scramble and shRNA vectors express GFP protein. (**B**) Quantification of blots in (A) showing that PCDH19 and alpha 1 levels, normalized on GAPDH, vary accordingly (see Supplementary Material, Table S3). (**C**) Quantification (± SEM) of synaptic markers (GABA_A_R alpha 1, GAD65/67, gephyrin, NCAD and GluA2/3) normalized to GAPDH from western blots of neurons infected as in (A) with scramble, shRNA and PCDH19-V5 (2X concentration). PCDH19 overexpression increases inhibitory markers expression with respect to the control condition, but not NCAD and GluA2/3 expression. GAD65/67 expression was significantly reduced by PCDH19 shRNA (one-way ANOVA, post-hoc Tukey’s test, *P < 0.05, **P < 0.01, ***P < 0.001; Supplementary Material, Table S4). (**D**) Biotinylation experiments using hippocampal cultured neurons infected at DIV8 with control shRNA (Scramble), shRNA and PCDH19-V5 (2X) and lysed at DIV12. (**E**) Quantification (± SEM) of the GABA_A_R subunits alpha 1 and 2 in whole lysate normalized to GAPDH (input) and the surface fraction normalized to the surface transferrin receptor (TfR) (biotinylated). The surface amount of both alpha 1 and 2 subunits varied according to the PCDH19 expression levels, with alpha1 showing the greatest variation (one-way ANOVA, post-hoc Tukey’s test, *P < 0.05, **P < 0.01, ***P < 0.001; Supplementary Material, Table S5).

To achieve this goal, we designed a short-hairpin (sh)RNA specific for PCDH19 (Supplementary Material, Fig. S1 A and B; Table S2). The shRNA and the human PCDH19 cDNA (PCDH19-V5), lentivirally expressed individually or in combination (rescue condition), allowed fine tuning of the PCDH19 expression level in hippocampal neurons (Fig. 3A and B; Supplementary Material, Table S3). Hippocampal neurons cultured for one week [from days *in vitro* (DIV) 1 to DIV8] in the presence of reduced or enhanced levels of PCDH19 showed altered expression of synaptic markers. Differences were observed in the inhibitory synaptic markers analysed, namely the GABA_A_R alpha 1 subunit, the glutamic acid decarboxylase (GAD) enzymes GAD65/67 and the scaffolding protein of inhibitory synapses gephyrin. Changes in the expression of the AMPA receptor (AMPAR) subunit GluA2/3 were not statistically significant. Neuronal cadherin (NCAD), which has been reported to interact with PCDH19 (8,16) and is expressed at both inhibitory and excitatory synapses early in synaptogenesis (17), showed different expression levels only between the shRNA and overexpressed conditions. The effect of PCDH19 downregulation was most strongly observed on the GAD65/67 expression level, which was reduced, while PCDH19 overexpression enhanced the expression of all the inhibitory synaptic markers analysed (Fig. 3B and C; Supplementary Material, Tables S3 and S4).

In neurons infected later and for a shorter period (4 days, from DIV8 to DIV12), we detected no significant changes in the total amount of GABA_A_R alpha 1, but PCDH19 down- or upregulation still bidirectionally modulated the surface levels of alpha 1. Consistently with the PCDH19 binding capability evaluated by GST pull-down (Fig. 2G), similar results were obtained for the GABA_A_R alpha 2 subunit. However, while PCDH19 downregulation reduced surface alpha 1 by nearly 70%, it reduced alpha 2 by approximately 45% (Fig. 3D and E; Supplementary Material, Table S5).

**Figure 4.**
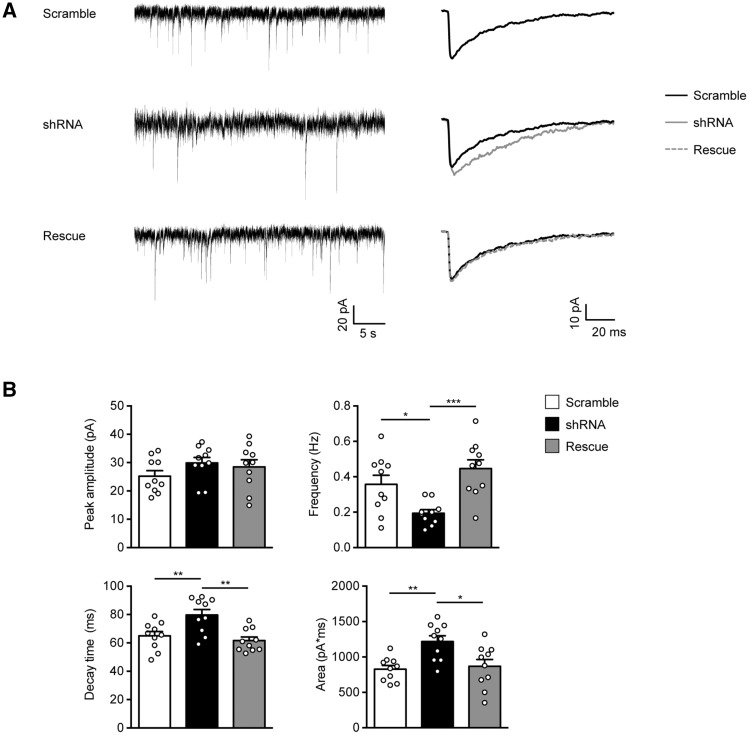
PCDH19 shRNA-mediated downregulation affects the miniature inhibitory post-synaptic currents (mIPSCs) frequency and kinetics. (**A**) Representative mIPSC recording (left) and population averages (right) from primary hippocampal pyramidal neurons expressing either PCDH19 control shRNA (Scramble), shRNA or shRNA plus PCDH19-V5 (Rescue). Neurons were transfected at DIV11 and recorded at DIV15. (**B**) Summary of mIPSC data (± SEM) showing that PCDH19 downregulation reduced the mIPSC frequency. Furthermore, mIPSCs of shRNA-expressing neurons were slower, as demonstrated by the increased decay time and area. Peak amplitude was not significantly affected by PCDH19 downregulation (one-way ANOVA, post-hoc Tukey’s test, *P < 0.05, **P < 0.01, ***P < 0.001; Supplementary Material, Table S6).

To evaluate the functional consequences of the GABA_A_R decrease on the neuronal surface, we measured miniature inhibitory postsynaptic currents (mIPSCs) from neurons transfected with scramble, shRNA and shRNA plus PCDH19-V5 (Rescue). Notably, mIPSCs of shRNA-expressing neurons were less frequent and slower, as inferred from the increased decay time and area. The rescue condition restored mIPSC parameters to control levels (Fig. 4A and B; Supplementary Material, Table S6).

Although mIPSC frequency changes might be due to both pre- and post-synaptic defects, we could ascribe the observed phenotype to the post-synapse, as we recorded transfected neurons innervated mainly by untransfected PCDH19-positive cells due to the low efficiency of the calcium phosphate method. Furthermore, a reduced frequency of mIPSCs was consistent with less GABA_A_R on the cell surface and at synapses, as suggested by the biotinylation assays. The slowing down of kinetic currents following PCDH19 downregulation suggested an altered composition of GABA_A_Rs. In particular, mIPSC prolongation occurs in the absence of the alpha 1 subunit (18,19).

Altogether, our data indicate that the molecular composition of GABAergic synapses in cultured hippocampal neurons is affected by the PCDH19 expression level and disclose a role for PCDH19 in regulating the surface availability of GABA_A_Rs and most likely their subunit composition.

### PCDH19 downregulation *in vivo* affects migration as well as morphological maturation of hippocampal neurons, and increases seizure susceptibility

**Figure 5.**
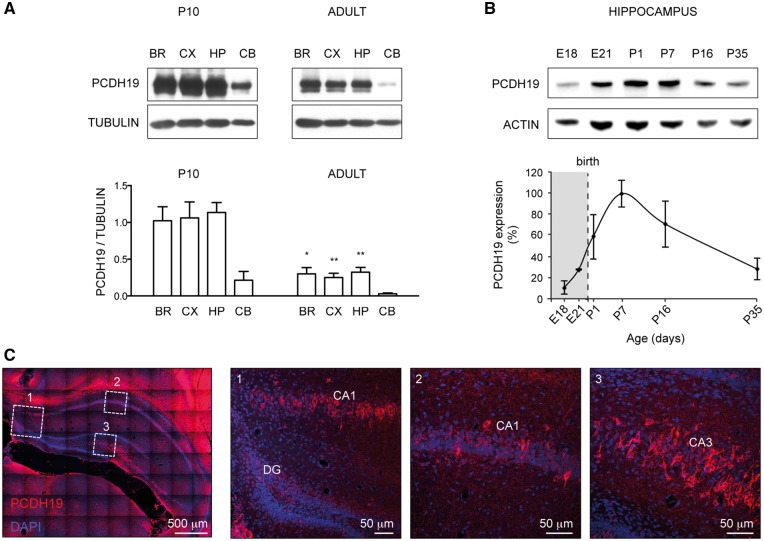
PCDH19 is highly expressed during early postnatal development in the rat hippocampus. (**A**) Western blots (top) and relative quantification (± SEM, bottom) showing PCDH19 expression in different brain areas (BR, all brain regions except cerebellum; CX, cortex; HP, hippocampus; CB, cerebellum) at postnatal day (P)10 and in adult (2–2.5 months old) rats (Student’s *t*-test, *P < 0.05, **P < 0.01; Supplementary Material, Table S7). (**B**) Western blot (top) and relative quantification (± SEM, bottom) showing the temporal expression of PCDH19 in lysates of rat hippocampi at different embryonic (E) and postnatal (P) days (Supplementary Material, Table S8). (**C**) Representative IHC images of PCDH19 in P10 rat hippocampus. PCDH19 is shown in red, the nuclear marker DAPI in blue and the merge in purple. Magnification insets 1, 2 and 3 show dentate gyrus (DG), CA1 and CA3 regions with PCDH19-positive pyramidal neurons.

To infer how the PCDH19 mutation might impact the functions of the GABAergic system *in vivo* during development, we first investigated the temporal expression of PCDH19 in rat brain. In both cortex and hippocampus, where PCDH19 is highly expressed, PCDH19 protein level was significantly higher at postnatal day (P)10 compared with the adult stage (Fig. 5A; Supplementary Material, Table S7). In particular, a time course in hippocampus from embryonic day (E)18 to P35 revealed a gradual increase in PCDH19 that peaked around the first postnatal week and successively declined (Fig. 5B; Supplementary Material, Table S8). At P10, PCDH19 protein was detectable in the pyramidal neurons of CA1 and CA3 regions, but at reduced levels in the dentate gyrus (DG) (Fig. 5C; Supplementary Material, Fig. S2), consistent with previously reported PCDH19 mRNA levels (20).

**Figure 6.**
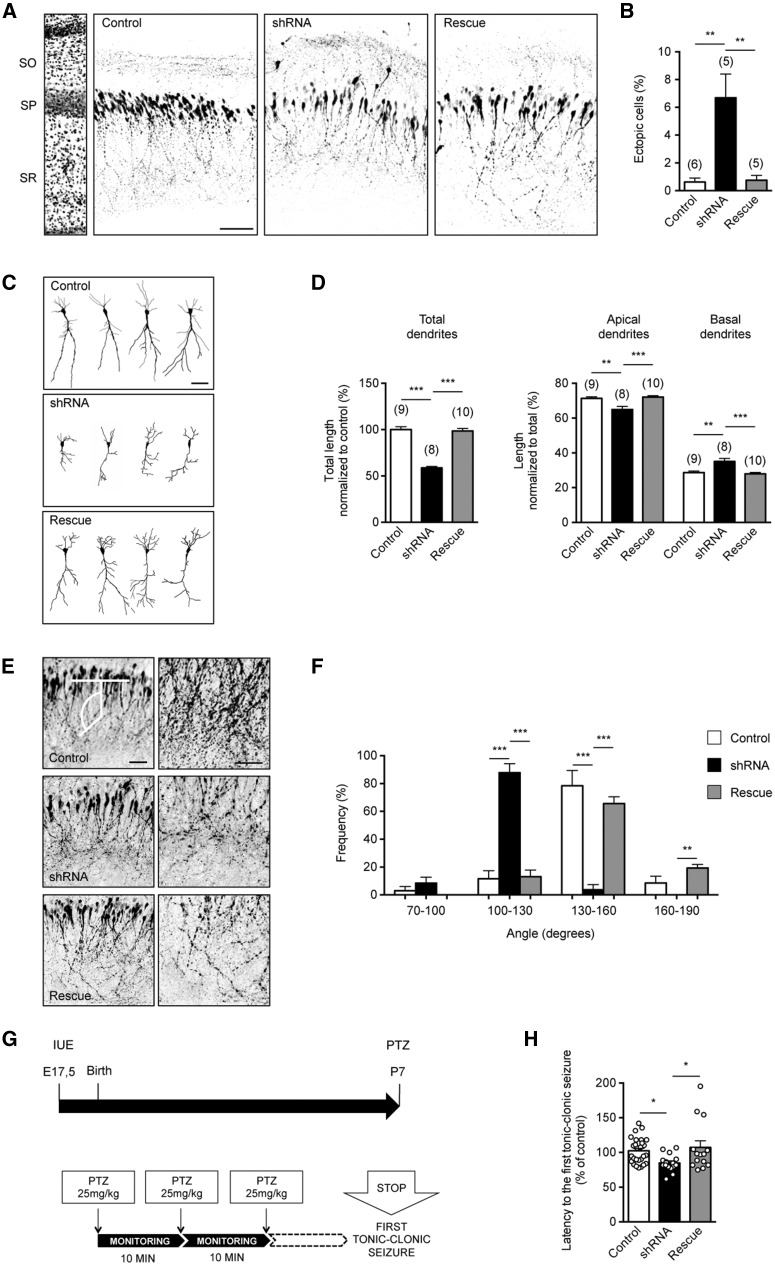
PCDH19 downregulation in rats affects the migration, morphological maturation and orientation of hippocampal neurons and increases seizure susceptibility. (**A**) Confocal images of GFP fluorescence in coronal sections of rat hippocampus at P7 after in utero transfection (at E17.5) with pRNAT-U6.3/Hygro empty vector (Control), PCDH19 shRNA or shRNA plus PCDH19-V5 (Rescue). Slices were counterstained with the nuclear marker Hoechst for visualization of hippocampal layers (left). Scale bar, 50 μm. SO, *stratum oriens*; SP, *stratum pyramidale*; SR, *stratum radiatum*. (**B**) Quantification of the number of ectopic cells (± SEM) in control, shRNA and rescue conditions. Numbers are expressed as a percentage of the ectopic cells normalized to the total number of fluorescent cells in the same section. Asterisks: statistically significant difference (Student’s *t*-test, **P < 0.01; Supplementary Material, Table S9). In parenthesis: total number of animals (1 slice/animal). (**C**) Representative reconstructions of neurons electroporated with the control vector, PCDH19 shRNA or shRNA plus PCDH19-V5 (Rescue). Scale bar, 50 μm. (**D**) Quantification of the average total dendritic length (normalized to controls, ± SEM) (left panel) and of the average length of apical or basal dendrites (normalized to the total length of all dendritic processes in the same neuron, ± SEM) (right panel) of neurons as in A. Asterisks: statistically significant difference (Student’s *t*-test, **P < 0.01, ***P < 0.001; Supplementary Material, Table S9). In parenthesis, total number of cells (3 animals/group). (**E**) Confocal images of GFP fluorescence in pyramidal neurons electroporated with the control vector, PCDH19 shRNA or shRNA plus PCDH19-V5 (Rescue) (left) and higher magnification images (right) showing the orientation of the apical dendrites in the SR. The white lines in the control image show the criteria utilized to calculate the angle of the processes. Scale bar, 50 μm. (F) Quantification of the frequency of the angles (binned in four groups) of the first main branching of the apical dendrite in cells as in A. Data are presented as a percentage (± SEM) of all cells in the control, shRNA or rescue condition (one-way ANOVA, post-hoc Holm-Sidak, **P < 0.01, ***P < 0.001; 3–4 animals/group; Supplementary Material, Table S9). (**G**) Schematic graph of the experimental protocol. (H) Quantification of the time latency to induce the first generalized tonic-clonic seizure in P7 pups electroporated into the hippocampus with control vector, PCDH19 shRNA or shRNA plus PCDH19-V5 (Rescue). The circles represent data points from single animals, and histograms represent the average. Numbers are expressed as percentages normalized to controls (± SEM) (one-way ANOVA, Holm-Sidak *post hoc* test, *P < 0.05; Supplementary Material, Table S10).

The rising phase of PCDH19 expression temporally overlaps with the migration and morphological maturation of hippocampal neurons, which relay on GABAergic signaling (21). Thus, if PCDH19 is required for GABAergic transmission during hippocampal development *in vivo*, PCDH19 loss of function might impair these processes. To verify this hypothesis, we delivered PCDH19 shRNAs together with GFP into the hippocampus of E17.5 rat embryos via *in utero* electroporation (IUE), and analysed transfected pyramidal neurons at P7. Compared with neurons from control rats expressing GFP alone, approximately 7% of shRNA-expressing CA1 neurons displayed ectopic localization, indicating altered migration (Fig. 6A and B; Supplementary Material, Table S9). Furthermore, PCDH19 downregulation affected neuronal arborization, as inferred from the reduction of total dendritic length and increased proportion of basal dendrites at the expense of apical ones (Fig. 6C and D; Supplementary Material, Table S9). Finally, the spatial orientation of apical dendrites in the *stratum radiatum* was also affected (Fig. 6E and F; Supplementary Material, Table S9). The rescue condition (shRNA plus PCDH19-V5, Rescue) restored migration and morphological parameters to control levels (Fig. 6A–F; Supplementary Material, Table S9).

Aberrant patterns of brain development that impair neuronal migration and circuitry formation are often associated with epilepsy (22) and recently cortical malformations have been identified in PCDH19-FE patients (23). This knowledge prompted us to evaluate susceptibility to pharmacologically induced seizures in P7 pups in which PCDH19 was downregulated in the hippocampus. Notably, the latency to induce the first generalized tonic-clonic seizure was significantly reduced in shRNA-expressing animals, while the expression of PCDH19 rescued the phenotype (Fig. 6G and H; Supplementary Material, Table S10).

Altogether, our data indicate that PCDH19 downregulation during neuronal development *in vivo* affects the migration as well as the morphological maturation of pyramidal hippocampal neurons, and increases seizure susceptibility.

## Discussion

In this study, we disclose a previously unknown role of PCDH19 in regulating GABAergic transmission in an attempt to provide insights into the phenotype of PCDH19-FE, characterized by epilepsy and intellectual disability.

We started by characterizing a newly identified interaction between PCDH19 and the alpha subunits of GABA_A_R. Based on our colocalization analysis, and in accordance with other protocadherins’ expression pattern (24–26), PCDH19 does not appear to be a constitutive component of the GABAergic synapses nor an ubiquitous binding-partner of GABA_A_Rs. However, GST pull-down assays indicate that PCDH19 associates with different alpha subunits, suggesting that PCDH19 is able to bind heterogeneous GABA_A_Rs, located both at synapses and at extrasynaptic sites. Since another protocadherin, Pcdh-γC5, has been previously reported to interact with the GABA_A_R subunit gamma 2 and to stabilize some GABAergic synapses (27), it is possible that different protocadherins might interact with specific pools of GABA_A_Rs and differentially modulate GABAergic transmission.

Despite PCDH19-GABA_A_Rs partial colocalization, the modulation of PCDH19 expression levels affected the composition and functioning of GABAergic synapses.

In particular, our data suggest that PCDH19 exerts a pro-wiring effect in neurons during the first week of *in vitro* development, as inferred from the expression of pre- and postsynaptic inhibitory markers. At a later time point (DIV8–12), PCDH19 downregulation or overexpression bidirectionally affected the surface expression of GABA_A_R alpha subunits, especially alpha 1, possibly by regulating GABA_A_R intracellular trafficking. Consistently, neurons in which PCDH19 has been downregulated, displayed mIPSCs with reduced frequency and slower decay kinetic, which is a hallmark of synapses lacking alpha 1 (18,19). Alpha 1 depletion at synapses is expected to change the pharmacological properties of GABA_A_Rs because this subunit is responsible for their enhanced sensitivity to neurosteroids and benzodiazepines (28,29). Interestingly, it has been proposed that deficiency of neurosteroids may contribute to PCDH19-FE etiology (30) and the treatment with ganaxolone, a synthetic analog of allopregnanolone, was able to reduce seizure in PCDH19-FE patients (31). Based on our data, we hypothesize that PCDH19-FE syndrome might be due, not only to a deficiency of positive modulators of the GABA_A_R, i.e. of neurosteroids, but also to a defect of the GABA_A_R itself. Furthermore, the recruitment of the alpha 1 subunit at synapses in early postnatal life promotes a more precise setting of the temporal window for input integration and neuronal network synchronization (12,18,19,32–34). Hence, the reduction of GABA_A_Rs, especially those containing alpha 1, might alter neuronal circuit formation during brain development.

The highest expression levels of PCDH19 in rats extend from the perinatal period to approximately puberty (P32–35), with a peak around the first postnatal week. In this period, the GABA neurotransmitter exerts a trophic influence on developing neurons (12). Thus, we verified whether PCDH19 downregulation *in vivo*, from the late embryonic development to the first postnatal week, might compromise neuronal migration and arborization.

PCDH19 has been previously shown to impair cell migration in zebrafish (35) and to enhance the migration of neuronal progenitors *in vitro*, even though no gross morphological defects were observed in the PCDH19 knockout (KO) mouse (36). In addition, cortical malformations have been recently identified in epileptic patients with *PCDH19* mutations (23). Concerning neuronal arborization, protocadherins are emerging as key regulators of dendrites development (37), and PCDH19 harbors a putative binding site for the WAVE regulatory complex (WRC) that controls actin cytoskeletal dynamics (38). Even though layer Va cortical neurons of the PCDH19 KO mouse display normal neurites (26), neurons of other brain regions were not analysed. Our data from IUE experiments support the involvement of PCDH19 in both the migration and dendritic branching of the CA1 hippocampal neurons. Indeed, a significant proportion of PCDH19 shRNA-expressing neurons displayed an ectopic location and their dendrites exhibited an altered spatial orientation and reduced arborization. Notably, these structural defects were associated with an increased susceptibility to pharmacologically induced seizures, indicating enhanced excitability of the neuronal network.

Altogether, these structural and functional defects might be ascribed to an altered functioning of the GABAergic system, especially with consideration of the ability of PCDH19 to associate with both synaptic and extrasynaptic GABA_A_Rs, these last being responsible for the trophic action of GABA (12). However, it is likely that more than one mechanism contributes to the phenotype observed *in vivo*. In particular, both the homophilic trans adhesive properties of PCDH19 (5) and its interaction with cytoskeleton regulators such as the WRC (38) must be taken into account, since they provide additional mechanisms to drive neuronal migration and development. Hence, the next challenge will be to unravel the impact of PCDH19 loss of function on cellular adhesion, cytoskeleton dynamics and GABAergic signaling, and consequently the relative contribution of these pathways in the development of neuronal circuits and in PCDH19-FE etiology.

In conclusion, the newly reported role of PCDH19 in regulating GABA_A_R-mediated currents is expected to impact both on the developmental processes that rely on GABAergic transmission and on the inhibitory/excitatory balance of the brain, and provides a new pathogenic mechanism for the PCDH19-FE syndrome.

## Materials and Methods

### cDNA and shRNA constructs

For a list of plasmids, cloning procedures and shRNA sequences, see the supplementary experimental procedures.

### Cell culture, transfection and infection

HEK293T and COS-7 cells were maintained in DMEM supplemented with 10% FBS, 1% GlutaMAX, 1% penicillin and 1% streptomycin. Cells were transiently transfected using a JetPEI Transfection Kit (Polyplus Transfection) according to the manufacturer’s instructions. After 48 h, the cells were either fixed for ICC or lysed for western blot experiments. HEK293FT were maintained in D-MEM (high glucose) supplemented with 10% fetal bovine serum (FBS), 0.1 mM MEM non-essential amino acids, 6 mM L-glutamine, 1 mM MEM sodium pyruvate, 1% pen-strep and 500 μg/ml geneticin and were used for the production of genetically modified lentiviruses as previously described (39,40). Briefly, cells at 50–70% confluence were transfected with second-generation lentiviral transfer vectors using the calcium phosphate method, and viral particles were concentrated from the cell medium 48 h later by ultracentrifugation. Primary hippocampal rat neurons were cultured using homemade B27 as previously described (41) except for a final medium concentration of 2.5 µg/ml of apo-Transferrin (Sigma) instead of 5 µg/ml of holo-Transferrin. Neurons were transfected by either the calcium phosphate method (electrophysiological recordings) or infected with lentiviral particles (biochemical assays) as indicated.

### ICC, acquisition and analysis of images

Cultured hippocampal neurons or HEK293T cells were fixed in 4% paraformaldehyde/4% sucrose for 8–10 min at room temperature (RT). Primary and secondary antibodies were applied in gelatin detergent buffer (GDB: 30 mM phosphate buffer at pH 7.4 containing 0.2% gelatin, 0.5% Triton X-100 and 0.8 M NaCl) at RT for 2 h and 1 h, respectively. The following primary antibodies were used: anti-V5 rabbit (Millipore, 1: 600); anti-myc mouse (Invitrogen, 1: 200); anti-PCDH19 rabbit (Bethyl Laboratories Inc., 1: 300); anti-GABA_A_R alpha 1 mouse (NeuroMab, 1: 300). Secondary antibodies were used at a concentration of 1: 300 (anti-mouse and anti-rabbit Alexa Fluor 488, Life Technologies; anti-mouse Alexa Fluor 555, Life Technologies; anti-rabbit 649 DyLight, Jackson Immnunoresearch).

ICC images were acquired with an LSM 510 Meta confocal microscope (Carl Zeiss, Italy) with a 60X oil-immersion objective at 1024 × 1024 pixel resolution. If not otherwise stated, image data were Z series projections of 6–8 images collected at depth intervals of 0.75 μm. Colocalization analysis was performed on single Z-sections using the ImageJ JaCop plug-in (42). Images were thresholded according to their gray level histogram (mode plus six times the standard deviation) and watershedded. In particular, Pearson’s (R), Manders’ (M1 and M2) and Van Steensel's cross correlation function (CCF) were evaluated.

### Biochemistry

Cell lysis and brain tissue homogenization was performed in modified RIPA buffer (50 mM TRIS-HCl, 200 mM NaCl, 1 mM EDTA, 1% NP40, 1% Triton X-100, pH 7.4, protease inhibitor cocktail).

#### CoIP

Soluble extracts from cells (HEK293T or primary hippocampal neurons) or rat brain tissue (cortex plus hippocampus) were incubated overnight at 4°C with the following mouse antibodies: anti-myc (10 μg/ml, Invitrogen), anti-PCDH19 (10 μg/ml, AbCAM) or non-immune control IgG. Protein A agarose beads (Invitrogen) were added and incubation continued for 2 h. The bead-antibody complexes were collected by centrifugation and washed 4–5 times with lysis buffer plus protease inhibitors, re-suspended in SDS sample buffer and boiled for 5 min before SDS/PAGE.

#### GST pull-down

GST fusion proteins were produced in *Escherichia coli* BL21 strain and purified and immobilized on glutathione-Sepharose 4B beads (GE Healthcare) according to standard procedures. HEK293T lysates or rat brain (cortex plus hippocampus) homogenates were incubated overnight at 4°C with GST fusion protein-bead complexes, washed 4–5 times in lysis buffer and re-suspended in SDS sample buffer and boiled. Samples were separated by SDS/PAGE followed by western blotting with the appropriate antibodies.

#### Biotinylation assay

Proteins on the plasma membrane of cultured hippocampal neurons were biotinylated by incubating cells with membrane-impermeable sulfo-NHS-SS-biotin (0.3 mg/ml, Pierce) for 5 min at 37°C. The cells were then washed with Tris-buffered saline (TBS: 10 mM Tris, 150 mM NaCl, pH 7.4) supplemented with 0.1 mM CaCl_2_, 1 mM MgCl_2_ and 50 mM glycine at 37°C. A second wash was performed on ice with the same solution prepared without glycine. The neurons were then lysed in a buffer composed of 50 mM Tris–HCl, pH 7.4, 1 mM EDTA, 150 mM NaCl, 1% SDS and protease inhibitors. The lysates were boiled for 5 min and incubated overnight with streptavidin-conjugated beads (Invitrogen) at RT. Biotinylated protein-bead complexes were collected by centrifugation, washed 4–5 times in lysis buffer, re-suspended and boiled in SDS sample buffer before SDS/PAGE. For details regarding the western blot protocol and a list of antibodies, see supplementary experimental procedures.

### Electrophysiology

Patch clamp recordings were performed in whole-cell configuration on primary hippocampal neurons at DIV15 using an Axopatch 200B Amplifier and pClamp 10.2 software (Molecular Devices). Traces were sampled at 10 KHz using a Digidata 1322A acquisition Interface (Molecular Devices) and filtered at 1 KHz. Borosilicate glass pipettes (GB150F-8P, Science Products) with a resistance of 4–6 MΩ were filled with an intracellular solution containing the following (in mM): 140 CsCl, 2 MgCl_2_, 1 CaCl_2_, 10 EGTA, 10 HEPES-CsOH, 2 ATP (disodium salt). Extracellular solution contained the following (in mM): 140 NaCl, 3 KCl, 1.2 MgCl_2_, 2 CaCl_2_, 10 glucose, 10 HEPES (pH 7.4). Blockers of excitatory synaptic transmission (3 mM kynurenic acid) and of sodium channels (0.5 μM lidocaine) were included in the extracellular solution. mIPSC were recorded in gap-free voltage clamp mode, holding the membrane potential at -60 mV (Vh = -60 mV), for a period of 5–10 min.

### Animals, neuronal cultures, IUE and PTZ seizure test

All animal care and experimental procedures were performed in accordance with the Italian Institute of Technology (IIT) and CNR licensing and were approved by the Italian Ministry of Health (authorization no. 1274/2015-PR and 100/2016).

Primary hippocampal neurons were prepared from rat embryos of either sex at E18 (Charles River, Italy) as described by Bassani *et al.* (2012) (43). Neurons were plated on coverslips coated with poly-D-lysine at a density of 75.000/well for ICC or 150.000/well for biochemistry.

IUE was performed as previously described (44). Briefly, E17.5 timed-pregnant Sprague Dawley rats (Harlan Italy SRL) were anesthetized with isoflurane (induction, 3.5%; surgery, 2.5%), and the uterine horns were exposed by laparotomy. DNA [4–6 μg/μl in water; pRNAT-U6.3/Hygro empty vector (Control), PCDH19 shRNA (shRNA), or shRNA plus PCDH19-V5 (Rescue)] together with the dye Fast Green (0.3 mg/ml; Sigma) was injected (5–6 μl) through the uterine wall into the lateral ventricles of each embryo. After injection, the head of the embryo was placed between two tweezer-type circular electrodes and an additional third one. For the electroporation protocol, five electrical pulses (amplitude, 50 V; duration, 50 ms; intervals, 150 ms) were delivered with a square-wave electroporation generator (CUY21EDIT; Nepa Gene). After electroporation, the uterine horns were returned into the dam’s abdominal cavity, and normal embryos development was resumed. For the PTZ seizure test, P7 pups were separated from the mother and placed in experimental boxes after intraperitoneal injection of pentylenetetrazol (PTZ, 25 mg/kg). The injection was repeated every 10 min until the appearance of the first tonic-clonic seizure.

### Acquisition and analysis of confocal and neurolucida images from brain slices

Brains from P7 pups were fixed by transcardial perfusion of 4% PFA in PBS. Fixed samples were cryopreserved in 30% sucrose. Brains were then frozen and sectioned coronally into 80-μm-thick slices using a microtome-refrigerator (Microm HM 450 Sliding Microtome equipped with Freezing Unit, Thermo Scientific, Italy). For migration analysis, images from the coronal sections were counterstained with Hoechst and acquired on a confocal laser-scanning microscope (TCS SP5; Leica Microsystems, Italy) equipped with a 10X immersion objective [numerical aperture (NA) 0.3]. Confocal images (15-µm-thick Z-stacks) were acquired, and Z-series were projected into two-dimensional representations. The contrast of the images was adjusted to enhance the fluorescence of the cell bodies while attenuating the signal from neuronal processes to facilitate cell counting. For high-magnification images of cell morphology, 50-μm-thick Z-stacks from the CA1 region were acquired with a 63X immersion objective (NA 1.4), and Z-series were projected into two-dimensional representations. Cell reconstructions were performed with Adobe Photoshop CS6 software. To quantify the dendrites, two-three confocal images/slice were acquired. To quantify cell processes, high-magnification images of cells were analysed with the NeuronJ plugin of the ImageJ software. Immunohistochemistry (IHC) was performed on 20-μm-thick brain slices. For details regarding the IHC staining and image acquisition and processing, see supplementary experimental procedures.

### Statistical analysis

Statistical analysis was performed with GraphPad Prism software. The significance of the data was assessed using the tests indicated in the figure legends. All data are presented as the means ± SEM. The results were considered statistically significant when *P* < 0.05. For the biotinylation experiments, the data were obtained from 2 to 6 independent neuronal preparations. For all the other *in vitro* experiments, data were obtained from at least three independent neuronal preparations. For values and details, see Supplementary Material, Tables S1–S10.

## Supplementary Material

Supplementary Material is available at *HMG* online.

## Supplementary Material

Supplementary Figures and Tables
