# Supplementary material for: The female epilepsy protein PCDH19 is a new GABAAR-binding partner that regulates GABAergic transmission as well as migration and morphological maturation of hippocampal neurons
Source: Hum Mol Genet. 2018 Jan 17;27(6):1027–38. doi: 10.1093/hmg/ddy019 (PMC5886308; doi:10.1093/hmg/ddy019)
Supplement: Supplementary Figures and Tables [file suppl_material_and_figures_ddy019.pdf]

Supplemental Information

Supplemental Data

A

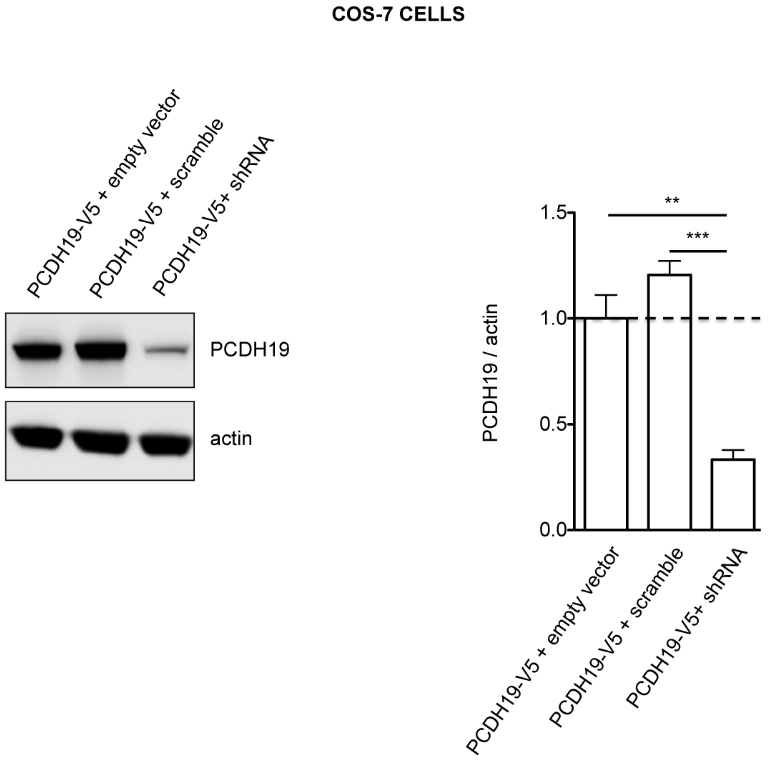

B

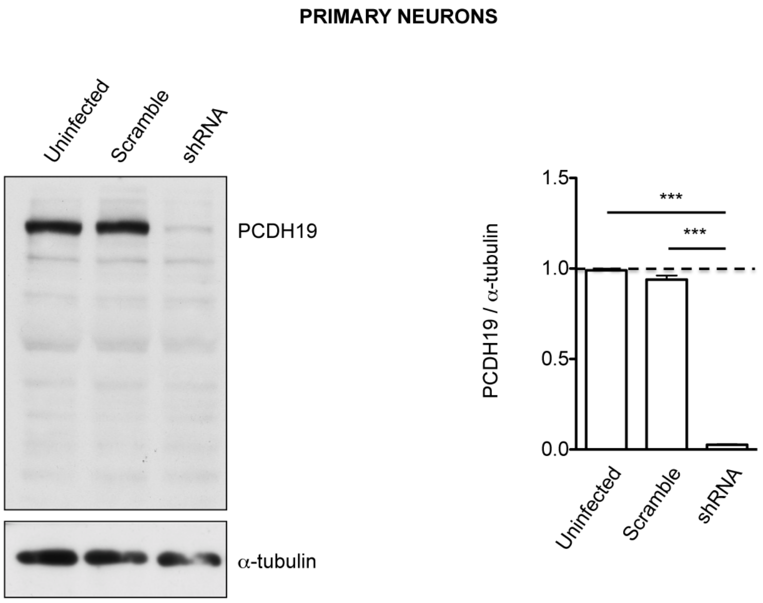

Figure S1 (related to Figure 3). Validation of PCDH19 shRNA

(A) Representative western blot of COS-7 cell lysates (left) and relative quantification (± SEM, right). Cells were transfected with human PCDH19 cDNA tagged with V5 (PCDH19-V5) plus empty vector (pRNAT-U6.3), control shRNA (Scramble) or PCDH19 shRNA. PCDH19 was normalized to actin and to PCDH19-V5 plus empty vector. The PCDH19 shRNA significantly downregulated PCDH19 expression (one-way ANOVA and post-hoc Tukey's test, ***$p < 0.001$, **$p < 0.01$; Table S2).

(B) Representative western blot of primary neurons that were uninfected or infected with lentiviruses expressing control shRNA (Scramble) or shRNA (left), and quantification of PCDH19 expression (± SEM, right). While the shRNA was partially resistant to human PCDH19 cDNA (PCDH19-V5), it reduced the level of rat PCDH19 to close to zero (one-way ANOVA and post-hoc Tukey's test, ***$p < 0.001$; Table S2).

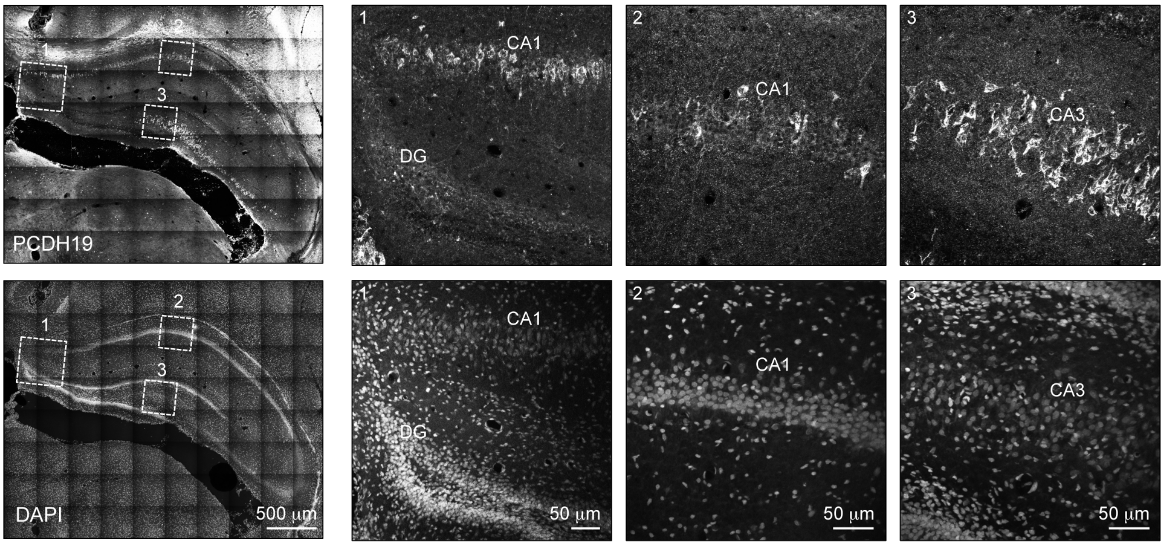

Figure S2 (related to Figure 5). Single channel images from IHC of PCDH19 and DAPI in hippocampus.

Representative images showing the expression of PCDH19 (top) and of the nuclear marker DAPI (bottom) in rat hippocampus at P10. Magnification insets 1, 2 and 3 show dentate gyrus (DG), CA1 and CA3 regions.

Table S1. Mean values, SEM and number of samples of data displayed in Figure 1D

**PCDH19-alpha1 colocalization**			
	Mean	SEM	N
R	0.622	0.013	12
M1	0.276	0.020	12
M2	0.299	0.031	12
**Pearson's coefficient (R)**			
ΔX (px)	Mean	SEM	N
-20	0.099	0.010	12
-19	0.106	0.010	12
-18	0.115	0.009	12
-17	0.125	0.009	12
-16	0.137	0.010	12
-15	0.151	0.010	12
-14	0.166	0.010	12
-13	0.184	0.010	12
-12	0.204	0.011	12
-11	0.227	0.012	12
-10	0.251	0.012	12
-9	0.280	0.013	12
-8	0.311	0.013	12
-7	0.347	0.013	12
-6	0.386	0.013	12
-5	0.429	0.013	12
-4	0.474	0.013	12
-3	0.524	0.013	12
-2	0.576	0.013	12
-1	0.626	0.013	12
0	0.662	0.013	12
1	0.634	0.013	12
2	0.587	0.013	12
3	0.533	0.014	12
4	0.482	0.014	12
5	0.435	0.014	12
6	0.391	0.014	12
7	0.352	0.014	12
8	0.317	0.014	12

9	0.286	0.013	12
10	0.258	0.013	12
11	0.232	0.013	12
12	0.208	0.012	12
13	0.188	0.011	12
14	0.170	0.011	12
15	0.155	0.011	12
16	0.141	0.010	12
17	0.130	0.010	12
18	0.119	0.010	12
19	0.109	0.010	12
20	0.101	0.010	12

Table S2. Mean values, SEM, number of samples and p values of data displayed in Figure S1

**shRNA validation**		Mean	SEM	N	p value (one-way ANOVA & Tukey's test)	
**COS-7 CELLS**						
PCDH19-V5 +	empty vector (A)	1.000	0.111	3	A vs. B	p > 0,05 (ns)
PCDH19-V5 +	scramble (B)	1.206	0.066	3	A vs. C	p < 0,01 (**)
PCDH19-V5 +	shRNA (C)	0.333	0.045	3	B vs. C	p < 0,001 (***)
**PRIMARY NEURONS**						
Uninfected (A)		1.000	0.010	2	A vs. B	p > 0,05 (ns)
Scramble (B)		0.939	0.023	2	A vs. C	p < 0,001 (***)
shRNA (C)		0.026	0.004	4	A vs. D	p < 0,001 (***)

Table S3. Mean values and number of samples of data displayed in Figure 3B

Markers expression	PCDH19		Alpha1	
	Value	N	Value	N
Scramble	1.000	1	1.000	1
shRNA	0.022	1	0.715	1
PCDH19-V5 (1)	2.014	1	1.548	1
PCDH19-V5 (2)	5.002	1	1.739	1
Rescue (1)	0.774	1	0.903	1
Rescue (2)	1.928	1	1.263	1

Table S4. Mean values, SEM, number of samples and p values of data displayed in Figure 3C

**Markers expression**	Mean	SEM	N	p value (one-way ANOVA & Tukey's *post hoc*)	
**Alpha1**					
Scramble	1.000	0.000	6	Scramble vs. shRNA	$p > 0.05$ (ns)
shRNA	0.529	0.196	6	Scramble vs. PCDH19-V5	$p < 0.05$ (*)
PCDH19-V5	2.898	0.835	4	shRNA vs. PCDH19-V5	$p < 0.01$ (**)
**GAD65/67**					
Scramble	1.000	0.000	7	Scramble vs. shRNA	$p < 0.01$ (**)
shRNA	0.677	0.077	7	Scramble vs. PCDH19-V5	$p < 0.05$ (*)
PCDH19-V5	1.279	0.090	5	shRNA vs. PCDH19-V5	$p < 0.001$ (***)
**Gephyrin**					
Scramble	1.000	0.000	4	Scramble vs. shRNA	$p > 0.05$ (ns)
shRNA	0.778	0.069	4	Scramble vs. PCDH19-V5	$p < 0.05$ (*)
PCDH19-V5	1.331	0.119	4	shRNA vs. PCDH19-V5	$p < 0.01$ (**)
**NCAD**					
Scramble	1.000	0.000	6	Scramble vs. shRNA	$p > 0.05$ (ns)
shRNA	0.877	0.072	6	Scramble vs. PCDH19-V5	$p > 0.05$ (ns)
PCDH19-V5	1.121	0.073	3	shRNA vs. PCDH19-V5	$p < 0.05$ (*)
**GluA2/3**					
Scramble	1.000	0.000	4	Scramble vs. shRNA	$p > 0.05$ (ns)
shRNA	0.895	1.243	4	Scramble vs. PCDH19-V5	$p > 0.05$ (ns)
PCDH19-V5	0.129	0.137	4	shRNA vs. PCDH19-V5	$p > 0.05$ (ns)

Table S5. Mean values, SEM, number of samples and p values of data displayed in Figure 3E

**Biotinylation**	Mean	SEM	N	p value (one-way ANOVA & Tukey's *post hoc*)	
**Alpha1 (INPUT)**					
Scramble	1.000	0.000	5	Scramble vs. shRNA	$p > 0.05$ (ns)
shRNA	1.042	0.112	5	Scramble vs. PCDH19-V5	$p > 0.05$ (ns)
PCDH19-V5	1.305	0.251	5	shRNA vs. PCDH19-V5	$p > 0.05$ (ns)
**Alpha1 (SURFACE)**					
Scramble	1.000	0.000	6	Scramble vs. shRNA	$p < 0.001$ (***)
shRNA	0.325	0.044	6	Scramble vs. PCDH19-V5	$p < 0.001$ (***)
PCDH19-V5	1.673	0.190	5	shRNA vs. PCDH19-V5	$p < 0.001$ (***)
**Alpha2 (INPUT)**					
Scramble	1.000	0.000	5	Scramble vs. shRNA	$p > 0.05$ (ns)
shRNA	0.994	0.034	5	Scramble vs. PCDH19-V5	$p > 0.05$ (ns)
PCDH19-V5	1.097	0.186	5	shRNA vs. PCDH19-V5	$p > 0.05$ (ns)
**Alpha2 (SURFACE)**					
Scramble	1.000	0.000	4	Scramble vs. shRNA	$p < 0.05$ (*)
shRNA	0.559	0.030	4	Scramble vs. PCDH19-V5	$p < 0.05$ (*)
PCDH19-V5	1.641	0.383	2	shRNA vs. PCDH19-V5	$p < 0.01$ (**)

Table S6. Mean values, SEM, number of samples and p values of data displayed in Figure 4B

**mIPSCs**	Mean	SEM	N	p value (one-way ANOVA & Tukey's *post hoc*)	
**Peak amplitude**					
Scramble	25.230	1.933	10	Scramble vs. shRNA	$p > 0.05$ (ns)
shRNA	29.869	1.954	10	Scramble vs. Rescue	$p > 0.05$ (ns)
Rescue	28.499	2.503	10	shRNA vs. Rescue	$p > 0.05$ (ns)
**Frequency**					
Scramble	0.357	0.051	10	Scramble vs. shRNA	$p < 0.05$ (*)
shRNA	0.194	0.021	10	Scramble vs. Rescue	$p > 0.05$ (ns)
Rescue	0.446	0.050	10	shRNA vs. Rescue	$p < 0.001$ (***)
**Decay time**					
Scramble	64.954	3.057	10	Scramble vs. shRNA	$p < 0.01$ (**)
shRNA	79.671	3.915	10	Scramble vs. Rescue	$p > 0.05$ (ns)
Rescue	61.650	2.525	10	shRNA vs. Rescue	$p < 0.01$ (**)
**Area**					
Scramble	827.062	53.159	10	Scramble vs. shRNA	$p < 0.01$ (**)
shRNA	1218.611	81.212	10	Scramble vs. Rescue	$p > 0.05$ (ns)
Rescue	869.038	95.767	10	shRNA vs. Rescue	$p < 0.05$ (*)

Table S7. Mean values, SEM, number of samples and p values of data displayed in Figure 5A

PCDH19 expression	P10			ADULT			p value (Student *t*-test)
	Mean	SEM	N	Mean	SEM	N	
BR	1.022	0.191	3	0.301	0.084	4	p=0.012 (*)
CX	1.061	0.216	3	0.251	0.058	4	P=0.009 (**)
HP	1.134	0.134	3	0.323	0.065	3	p=0.006 (**)
CB	0.214	0.119	3	0.030	0.011	4	p=0.127 (ns)

Table S8. Mean values, SEM and number of samples of data displayed in Figure 5B

PCDH19 expression	Mean	SEM	N
E18	14.140	3.256	3
E21	30.645	3.933	3
P1	60.632	18.046	3
P7	100.000	2.416	3
P16	72.202	18.326	3
P35	31.229	5.100	3

Table S9. Mean values, SEM, number of samples and p values of data displayed in Figure 6B, D, F

IUE	Mean	SEM	N (animals)	p value (Student *t*-test)
**ECTOPIC CELLS**				
Control	0.619	0.289	6	Control vs. shRNA p < 0,01 (**)
shRNA	6.691	1.705	5	Control vs. Rescue p > 0,05 (ns)
Rescue	0.754	0.352	5	shRNA vs. Rescue p < 0,01 (**)
**DENDRITES LENGTH**				
**Total dendrites**				
Control	100.000	3.194	3	Control vs. shRNA p < 0,001 (***)
shRNA	58.664	1.632	3	Control vs. Rescue p > 0,05 (ns)
Rescue	98.536	2.711	3	shRNA vs. Rescue p < 0,001 (***)
**Apical dendrites**				
Control	71.339	0.894	3	Control vs. shRNA p < 0,01 (**)
shRNA	64.958	1.722	3	Control vs. Rescue p > 0,05 (ns)
Rescue	72.08	0.779	3	shRNA vs. Rescue p < 0,001 (***)
**Basal dendrites**				
Control	28.661	0.894	3	Control vs. shRNA p < 0,01 (**)
shRNA	35.041	1.722	3	Control vs. Rescue p > 0,05 (ns)
Rescue	27.919	0.779	3	shRNA vs. Rescue p < 0,001 (***)
**DENDRITES ANGLE**				
**70-100**				
Control	3.030	3.030	3	Control vs. shRNA p > 0,05 (ns)
shRNA	8.465	4.330	3	Control vs. Rescue p > 0,05 (ns)
Rescue	0.000	0.000	4	shRNA vs. Rescue p > 0,05 (ns)
**100-130**				
Control	11.616	5.825	3	Control vs. shRNA p < 0,001 (***)
shRNA	87.830	6.501	3	Control vs. Rescue p > 0,05 (ns)
Rescue	13.125	4.718	4	shRNA vs. Rescue p < 0,001 (***)
**130-160**				
Control	78.355	11.054	3	Control vs. shRNA p < 0,001 (***)
shRNA	3.703	3.703	3	Control vs. Rescue p > 0,05 (ns)
Rescue	65.625	4.827	4	shRNA vs. Rescue p < 0,001 (***)
**160-190**				
Control	8.586	4.818	3	Control vs. shRNA p > 0,05 (ns)
shRNA	0.000	0.000	3	Control vs. Rescue p > 0,05 (ns)
Rescue	19.375	2.576	4	shRNA vs. Rescue p < 0,01 (**)

Table S10. Mean values, SEM, number of samples and p values of data displayed in Figure 6H

**IUE-PTZ**	Mean	SEM	N (animals)	p value (one-way ANOVA & Holm-Sidak)	
Control	100.000	4.099	27	Control vs. shRNA	$p < 0.05$ (*)
shRNA	84.778	3.162	15	Control vs. Rescue	$p > 0.05$ (ns)
Rescue	107.128	9.653	14	shRNA vs. Rescue	$p < 0.05$ (*)

Supplemental Experimental Procedures

cDNA and shRNA constructs

The human PCDH19 open reading frame with a C-terminus V5 tag was subcloned from PCDH19-V5 pcDNA-3.2nV5 (isoform 4, lacking amino acid 892 of the canonical isoform, accession number NM_001184880.1; gift from Prof. J. Gecz, University of Adelaide) into cFUW (Addgene) and pCAGGS-IRES-EGFP (gift from Dr. A. Contestabile, IIT). PCDH19-V5 in pCAGGS-IRES-EGFP was used in the experiments in Figure 6 and Supplemental Figure 1A. PCDH19-V5 in cFUW was used in all other experiments. The human PCDH19 sequence corresponding to amino acids 1-879 fused to tDIMER2 RFP was subcloned in cFUW to obtain PCDH19-Δ879. Portions of the human PCDH19 sequence were subcloned into pGEX 4.1T vector to obtain the following: CT (amino acids 700-1147), CT1 (700-890), CT2 (891-1148), CT3 (763-984). Similarly, $GABA_AR$ alpha1 TM3-4 sequences were subcloned into the pGEX 4.1T vector to obtain the following: TM (amino acids 334-

420), TM-A (334-355), TM-B (356-390), TM-C (391-420), TM-A1 (334-343), TM-A2 (344-355).

A PCDH19-specific shRNA (target sequence 5'-gagcagcatgaccaatacaat-3') and a control shRNA (Scramble, target sequence 5'-gctgagcgaaggagagat-3') were cloned into the pLVTHM vector (Addgene) and used for the experiments in Figures 3 and 4. The PCDH19 shRNA was also cloned in pRNAT-U6.3/Hygro (GenScript) and used for the experiments in Figure 6 and Supplemental Figure 1A. The shRNA has two mismatches with the human PCDH19 sequence (5'-gagcagca*c*gaccaatacaa*c*-3', mismatches in italics), conferring PCDH19-V5 partial resistance to the shRNA. Thus, PCDH19-V5 was used in combination with the shRNA for the rescue experiments. PCDH9 in the pIRES2 vector was a gift from Dr. Fengmin Lu (Peking University). The $GABA_AR$ subunits alpha1 with myc tags (myc-alpha1), beta2 and gamma2 in the pcDNA3 vector were a gift from Dr. M. Garret (University of Bordeaux).

Biochemistry

Western blotting and antibodies. The protein concentration of samples was estimated using the BCA kit (Pierce). Equivalent amounts of protein were applied to polyacrylamide gels and subjected to electrophoresis. Gels were blotted onto nitrocellulose membranes (Whatman), and equal protein loading was verified by brief staining with 0.1% Ponceau S solution. Membranes were blocked for 1 h in 5% milk in TBS (10 mM Tris, 150 mM NaCl, pH 8.0) plus 0.1% Tween- 20 and incubated overnight at 4 °C with primary antibodies against actin (rabbit, 1:5000; Sigma-Aldrich); myc (mouse, 1:1000; Invitrogen); V5 (rabbit, 1:2000; Millipore); PCDH9

(rabbit, 1:500; AbCam); PCDH19 (mouse, 1:500-1:1000; AbCam); PCDH19 (rabbit, 1:10000; Bethyl Laboratories, Inc.); GAD65/67 (rabbit, 1:500; Millipore); Gephyrin (rabbit, 1:500; Thermo Scientific); NCAD (rabbit, 1:1000; AbCam); GluA2/3 (rabbit, 1:2000; gift from Dr. C. Gotti); GAPDH (rabbit, 1:2000; Santa Cruz); GFP (rabbit, 1:2000; MBL International); Transferrin Receptor (TfR) (mouse, 1:1000; Life Technologies); alpha-tubulin (mouse, 1:50000; Sigma-Aldrich); $GABA_AR$ subunits: alpha1 (rabbit, Millipore; 1:1000); alpha2 (mouse, 1:500; Synaptic Systems), alpha3 (rabbit, 1:1000; Synaptic Systems), alpha5 (mouse, 1:1000; NeuroMab), beta1 (mouse, 1:1000; NeuroMab), beta3 (mouse, 1:1000; NeuroMab), gamma2 (rabbit, 1:1000; Synaptic Systems), and delta (mouse, 1:300; NeuroMab). Membranes were washed and incubated for 1 h at room temperature with peroxidase-conjugated anti-rabbit (1:5000; Bio-Rad) or anti-mouse (1:5000; Bio-Rad) antibodies. Stained membranes were developed using the Pierce ECL-detection kit or Super Signal West Pico chemoluminescent substrate. Bands were quantified by measuring the mean intensity of the band signal using ImageJ.

IHC staining and image acquisition

Coronal brain sections (20 μm) placed on polylysine slides (Thermo Scientific. Italy) were rinsed three times for 15 minutes each with PBS, and blocking solution (BSA, 3%; goat serum, 10%; Triton-X100, 0.4%. in PBS) was applied for 1 h at RT. Sections were incubated with primary antibody in blocking solution (rabbit anti-PCDH19 1:400. Bethyl Laboratories., Inc.) O/N at 4 °C, rinsed three times for 15 minutes each in PBS and incubated with secondary antibody (649 DyLight 1:2000,

Jackson Immnunoresearch) for 1 h at RT. After 3 washes in PBS, the sections were incubated with DAPI (1:1000, Thermo Scientific) for 10 min at RT to stain cell nuclei and washed 2 times with PBS. Stained sections were mounted using fluoromount (Sigma-Aldrich, Italy). IHC images were acquired with an LSM 510 Meta confocal microscope (Carl Zeiss). Images of distinct hippocampal areas were acquired using a 20X (zoom 1) or 40X (zoom 0.7) objective at 1024 x 1024 pixel resolution. Image data were Z series projections of 8-10 images collected at depth intervals of 0.75 μm. Images of the entire hippocampus were acquired using a 40X objective (zoom 0.7) at 512 x 512 pixel resolution with the Tile Scan function that allows the reconstruction of an overview image consisting of a number of tiled partial images (8 x 11 tiles).
